# QTL for induced resistance against leaf rust in barley

**DOI:** 10.3389/fpls.2022.1069087

**Published:** 2023-01-12

**Authors:** Andrea Matros, Adam Schikora, Frank Ordon, Gwendolin Wehner

**Affiliations:** ^1^ Julius Kühn Institute (JKI), Federal Research Centre for Cultivated Plants, Institute for Resistance Research and Stress Tolerance, Quedlinburg, Germany; ^2^ Julius Kühn Institute (JKI), Federal Research Centre for Cultivated Plants, Institute for Epidemiology and Pathogen Diagnostics, Braunschweig, Germany

**Keywords:** biotic stress resistance, Ensifer meliloti, AHL priming, Puccinia hordei, barley, leaf rust, GWAS, QTL

## Abstract

Leaf rust caused by *Puccinia hordei* is one of the major diseases of barley (*Hordeum vulgare* L.) leading to yield losses up to 60%. Even though, resistance genes *Rph*1 to *Rph*28 are known, most of these are already overcome. In this context, priming may promote enhanced resistance to *P. hordei*. Several bacterial communities such as the soil bacterium *Ensifer* (syn. *Sinorhizobium*) *meliloti* are reported to induce resistance by priming. During quorum sensing in populations of gram negative bacteria, they produce *N*-acyl homoserine-lactones (AHL), which induce resistance in plants in a species- and genotype-specific manner. Therefore, the present study aims to detect genotypic differences in the response of barley to AHL, followed by the identification of genomic regions involved in priming efficiency of barley. A diverse set of 198 spring barley accessions was treated with a repaired *E. meliloti* natural mutant strain *expR*+*ch* producing a substantial amount of AHL and a transformed *E. meliloti* strain carrying the lactonase gene *attM* from *Agrobacterium tumefaciens*. For *P. hordei* resistance the diseased leaf area and the infection type were scored 12 dpi (days post-inoculation), and the corresponding relative infection and priming efficiency were calculated. Results revealed significant effects (p<0.001) of the bacterial treatment indicating a positive effect of priming on resistance to *P. hordei*. In a genome‐wide association study (GWAS), based on the observed phenotypic differences and 493,846 filtered SNPs derived from the Illumina 9k iSelect chip, genotyping by sequencing (GBS), and exome capture data, 11 quantitative trait loci (QTL) were identified with a hot spot on the short arm of the barley chromosome 6H, associated to improved resistance to *P. hordei* after priming with *E. meliloti expR*+*ch*. Genes in these QTL regions represent promising candidates for future research on the mechanisms of plant-microbe interactions.

## 1 Introduction

Leaf rust caused by the biotrophic fungus *Puccinia hordei* is a serious disease of barley, leading to yield losses from 15–25% (Whelan et al., 1997) up to 62% (Park et al., 2015). Visual symptoms vary from small chlorotic lesions to large orange-brown pustules of about 0.5 mm in size, often surrounded by green leaf areas (Clifford, 1985). A major strategy in controlling leaf rust epidemics is breeding of resistant barley cultivars. While several resistance genes (*Rph*1 to *Rph*28, and *Rph*_MBR1012_) have been identified in recent years (Singh et al., 2015; Fazlikhani et al., 2019; Chen et al., 2021), most of these are already overcome. Breakdown of resistance by mutations in effector genes of the pathogen frequently lead to the occurrence of new virulent races (Park, 2003), and thus a rapid decrease of the number of effective *Rph* genes (Kavanagh et al., 2017). Other effective management practices include the application of chemical fungicides, which can cause negative environmental (Ballabio et al., 2018) and public health effects (Perlin et al., 2017) if applied incorrectly. In this regard, decision-based management strategies may help to reduce their environmental impact considerably in the coming years (Lázaro et al., 2021). However, to promote more sustainable agricultural systems establishing alternative strategies to manage leaf rust epidemics is of prime importance, besides the ongoing genetic mapping of resistance genes.

Novel approaches for sustainable and economical crop production under ever-changing climate conditions include microbiome-facilitated crop management strategies (Liu et al., 2020; Trivedi et al., 2021). Beneficial plant-associated microbes include predominantly rhizobacteria and fungi as well as viruses, actinomycetes, cyanobacteria, archaea (Mauch-Mani et al., 2017; Mahapatra et al., 2020). The barley-microbiome was reported to largely vary between the different plant organs, developmental stages, origin, as well as between genotypes (Yang et al., 2017; Alegria Terrazas et al., 2020; Bziuk et al., 2021). Reported positive effects of such interactions in barley include reduced virulence of plant pathogenic fungi (Wehner et al., 2019; Karlsson et al., 2021), higher tolerance to abiotic stress (Caddell et al., 2019; Yang et al., 2020), as well as plant growth promotion and nutrient uptake under adverse conditions (Trivedi et al., 2020; Ribaudo et al., 2020).

The activation of induced defense mechanisms by various stimuli, such as from pathogens, beneficial microbes, arthropods, as well as chemicals and abiotic cues, is generally regarded as priming (Mauch-Mani et al., 2017; Desmedt et al., 2021). Upon priming, plants respond stronger and faster to a biotic or abiotic stress factor, resulting in resistance and securing yield. The use of priming for enhancing resistance has a long tradition, and the phenomenon called “sensitization” or “preformed defense” was investigated and used since the 1930s (Chester, 1933; Starkey, 1958). In this respect, it has to be noted, that microbial priming-induced plant responses vary between species and depend on the composition of the soil-microbiome as well as on the genotype and vice versa (Jack et al., 2019; Morella et al., 2020; Alegria Terrazas et al., 2020; Si et al., 2021).

Various organic compounds, for instance salicylic acid (SA), benzothiadiazole, β-aminobutyric acid, and azelaic acid are known to induce priming (Conrath et al., 2002; Jung et al., 2009; Mauch-Mani et al., 2017). In addition, many recent reports are highlighting bacterial quorum sensing (QS) molecules as priming-inducers in plants. Related to plant-pathogen interactions, the best-studied QS molecules are *N*-acyl homoserine lactones (AHL), which are produced by numerous gram negative bacteria to communicate and to monitor the density of populations (Kaplan and Greenberg, 1985; Fuqua et al., 1994). Plants generally respond to short-chained AHL with modifications in growth, while long-chained AHL induce AHL-priming for enhanced resistance against pathogen infection (Shrestha et al., 2020).

In tomato plants, root colonization with AHL-producing *Serratia liquefaciens* MG1 and *Pseudomonas putida* IsoF lead to increased systemic resistance against the fungal leaf pathogen *Alternaria alternate*, likely in an SA- and ethylene-dependent manner (Schuhegger et al., 2006). The effect has been related to the four AHLs N-(3-oxo-hexanoyl)-L-homoserine lactone (3-oxo-C6-HSL), 3-oxo-C8-HSL, 3-oxo-C10-HSL, and 3-oxo-C12-HSL produced by *P. putida* IsoF (Gantner et al., 2006), and to the two AHLs butanoyl-homoserine lactone (C4-HSL) and 3-oxo-C6-HSL produced by *S. liquefaciens* MG1. Plants inoculated with the AHL-negative mutant strain *S. liquefaciens* MG44 exhibited a susceptible phenotype (Schuhegger et al., 2006).

Another rhizobacterium with known AHL-priming capacity is *Ensifer meliloti*, a naturally occurring gram negative soil bacterium (Teplitski et al., 2003; Fagorzi et al., 2020). By studying AHL-priming with *E. meliloti* as a model in the laboratory, different authors tested resistance against various pathogens, for instance *Pseudomonas syringae* in *Arabidopsis thaliana* (Zarkani et al., 2013; Shrestha et al., 2020), *Blumeria graminis* (Shrestha et al., 2019), *Puccinia hordei* in barley (Hernández-Reyes et al., 2014; Wehner et al., 2019; Shrestha and Schikora, 2020) and the pest *Pratylenchus penetrans* in soybean (Adss et al., 2021). Notably, barley genotypes showed different sensitivity to AHL-priming when treated with *E. meliloti*, indicated by diverse resistance responses of seven barley accessions to *P. hordei* infection (Wehner et al., 2019) and of eight barley accessions to *B. graminis* infection (Shrestha et al., 2019). These results suggest that AHL-priming is highly influenced by the genetic background of the respective accession, and strongly propose the utilization of priming-efficiency as a future breeding goal. However, the genetic background underlying the variability of AHL-priming-induced resistance in plants has not yet been investigated.

In order to map genetic loci underlying differences in response to AHL-priming, genome wide association studies (GWAS) are a powerful tool since high throughput marker technologies like the 9k iSelect chip (Comadran et al., 2012), genotyping by sequencing (Poland and Rife, 2012) or exome capture (EC) sequencing (Mascher et al., 2013) are available in barley. While family-based linkage analysis searches for associations within populations developed from bi-parental crosses, association mapping utilizes historic patterns of recombination that have occurred within a sample of individuals. Association mapping is based on the principle that over multiple generations of recombination, correlations only with markers tightly linked to the trait of interest will be present. As a predominantly inbreeding species, cultivated barley is an attractive target for association mapping as its genome contains extensive blocks of chromatin in linkage disequilibrium (Rostoks et al., 2006), providing a well-defined haplotype structure from which marker-trait associations can be identified (Cockram et al., 2008).

In this study, we aim to investigate the genetic background of resistance priming in barley. Thus, the objective is identifying barley varieties with an enhanced potential to respond to *E. meliloti* treatment with an increased resistance to a *P. hordei* infection by screening a set of barley genotypes with a diverse genetic background. A set of 198 spring barley accessions was challenged with either the 3-oxo-C14-HSL accumulating *E. meliloti* strain *expR*+*ch* or with the control *E. meliloti* strain *attM*, not being able to accumulate AHL (Zarkani et al., 2013). Responses after *P. hordei* infection were monitored and relative infection values were used as a target for GWAS. Identified primable accessions and QTL are discussed regarding the molecular mechanisms of AHL-priming and breeding applications.

## 2 Material and methods

### 2.1 Plant material, experimental design, and treatment

In order to determine the effects of priming in relation to an infection with *P. hordei* causing leaf rust, we made use of the potentially most diverse GWAS panel for barley comprising 200 spring barley accessions and landraces of worldwide origin from the spring barley IPK-SB224 panel, including the “Genobar” panel, (Haseneyer et al., 2010; Pasam et al., 2012). Our study panel consisted of 111 two-rowed and 83 six-rowed accessions from the Genobar panel and additional six barley lines, namely Barke, Morex, Steptoe, Golden Promise, Großklappige, and Roland, making it a total of 200 spring barley accessions of which 198 were genotyped (**Table S1** and **Table S2**, **Figure S1**).

**Figure 1:**
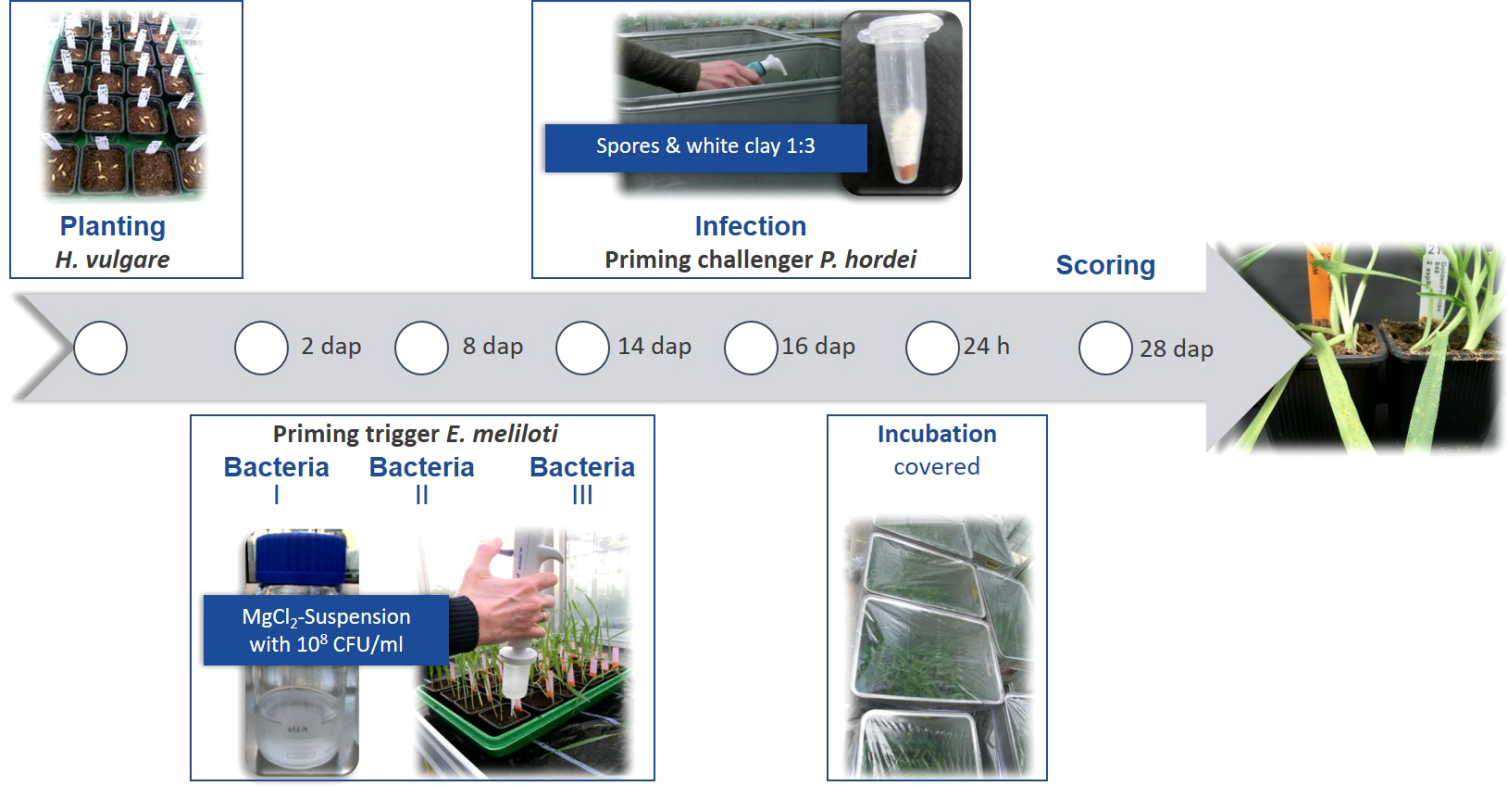
Experimental workflow of the priming treatment with *E. meliloti* followed by *P. hordei* infection and symptoms scoring. Days after planting – dap.

This panel was investigated in greenhouse pot experiments for three growing seasons (2017, 2018 and 2019) in a split plot design with three pots per accession. The experimental workflow is illustrated in **Figure 1**. After two days of germination on wet filter paper, seedlings were transferred to potting mix (Fruhstorfer Typ T) with three plants per pot (7x7x6 cm filled with 0.2 L of substrate) and cultivated in the greenhouse at night/day temperatures of 18°C/22°C with additional lighting for 10 h. For watering of pots to full water holding capacity, tap water was used, and the substrate was kept at similar moisture for all accessions and treatments during the course of the experiment. Two days after planting (dap) all accessions were treated either with the repaired *E. meliloti* natural mutant strain *expR*+*ch* (Rm2011 *expR*+*ch*) or with the transformed *E. meliloti* strain *attM* (pBBR2-*attM*). *ExpR*+*ch* is able to accumulate a large amount of the AHL 3-oxo-C14-HSL whereas *attM* was used as a control, carrying a lactonase gene from *Agrobacterium tumefaciens* and is therefore not able to accumulate AHL. Both bacteria strains were grown in tryptone yeast (TY) extract medium until the OD_600nm_ of 0.6 to 0.8. Bacterial cultures were centrifuged at 2,500 × g for 10 min and suspended in 10 mM MgCl_2_ (Zarkani et al., 2013). The bacteria solutions were applied three times in total. In fact, the substrate was treated with 3.5 ml of bacteria solution (OD_600nm_ of 0.1, corresponding to 10^8^ CFU/ml) each, at two, eight and 14 dap (Wehner et al., 2019) using a multi-dispenser pipette equipped with a 50 ml tip vessel. Inoculation of plants with the *P. hordei* virulent strain I-80 was performed at the three-leaf stage at 16 dap. Therefore, trays containing 24 pots each were placed in sealable containers. The leaf rust spores (25 mg) were mixed with white clay (1:3) and applied equally to the plants in the containers, using a manual powder spray bottle. Immediately after the inoculation, the containers were covered with foil to keep a moist atmosphere and the plants were stored for 24 hours without light. After removing the trays from the containers, plants were further cultivated in the greenhouse as stated above.

### 2.2 Phenotyping for relative infection and priming efficiency

The scoring was carried out 12 dpi (**Figure 1**) by estimating the diseased leaf area in percentage [%] (Moll et al., 2000) and the infection type (Levine and Cherewick, 1952). Thereof, the relative infection as well as priming efficiency were calculated according to Wehner et al. (2019).


Relative infection = 0.2 × ln (P. hordei %) + (P. hordei scores)

Priming efficiency = relative infection (attM) − relative infection (expR+ch)

Figures showing relative infection results were generated with the data analysis software JMP version 14 (https://www.jmp.com/en_gb/software/data-analysis-software.html). The barley accessions were clustered in primable and non-primable genotypes, by the difference of the relative infection between the priming treatments (= priming efficiency), applying a threshold for priming efficiency of >0.2 for primable ones and a significance level of p<0.05. Best linear unbiased estimates (BLUEs) of relative infection and priming efficiency for the set of accessions under investigation are provided in **Table S2**.

The descriptive statistics of the phenotypic data were carried out in the software “RStudio” version 3.4.2 (https://www.rstudio.com/products/rstudio/) using the R-packages “pastecs” and “agricolae”. The minimum (Min), the maximum (Max), mean, standard deviation (SD), coefficient of variation (CV, standard deviation divided by mean), least significant difference (LSD), and repeatability for relative infection with *P. hordei* for the 198 accessions and for three years experiments were calculated for *attM* as well as for *expR*+*ch* (**Table 1**). Repeatability of the experiments in the greenhouse was calculated based on the variance components of the accessions and the variance associated with the accession by year interaction implemented in the R-package “lme4”. Based on these data, an analysis of variances (ANOVA) was carried out over the total number of accessions in order to check for any significant effects for the accessions, the priming treatment and their interaction (**Table 2**). Thus, a linear mixed effects (lme) model by means of the R-package “nlme” was applied, using priming, accessions, and the interaction of both as fixed effects and the plant per pot, replication, and year as random effects.

**Table 1:** Descriptive statistics of relative infection with *P. hordei* for the 198 accessions treated with either the *attM* or *expR*+*ch* bacterial strain.

Priming	*attM*	*expR*+*ch*
Min	1.110	1.060
Max	1.690	1.440
Mean	1.490	1.310
SD	0.081	0.099
CV	0.054	0.075
LSD	0.004	0.010
r²	0.636	0.314

Minimum (Min), maximum (Max), mean, standard deviation (SD), coefficient of variation (CV, standard deviation divided by mean), least significant difference (LSD), and repeatability (r^2^) are listed.

**Table 2:** Analysis of variances (ANOVA) of relative infection with *P. hordei* for the 198 accessions.

Effect	F-value	P-value
Priming (αi)	306.06	<0.001
Accession (βj)	8.89	<0.001
(αβ)ij	3.33	<0.001

### 2.3 Genotyping and GWAS

For 198 accessions single nucleotide polymorphism marker data was gained (**Table S1**) derived from the Illumina 9k iSelect chip (SGS Trait genetics) following Comadran et al. (2012). In addition, for 175 accessions genotyping by sequencing (GBS) and for 155 accessions Exome Capture analyses (Mascher et al., 2013) were created. Thus, 5,764 iSelect markers, 17,653 GBS markers and 470,429 exome capture (EC) markers were used after filtering for<12.5% heterozygous SNPs, >5% minor allele frequency and<10% missing values. Genetic positions for all markers were extracted from the barley reference genome based on MorexV3 (Mascher et al., 2021) by means of the Barleymap software module “Locate by position” (https://floresta.eead.csic.es/barleymap/locate/), which was published by the international barley sequencing consortium (Cantalapiedra et al., 2015).

SNP markers from the iSelect Chip were used for calculation of the population structure in STRUCTURE (Pritchard et al., 2000). In this program, ten independent runs of Monte Carlo Markov chain with burn-in period of 500,000 were calculated to obtain the optimum k-value and the corresponding q-matrix. Two subpopulations clustered by row-type (**Figure S1**) were identified according to the method of Evanno et al. (2005). Furthermore, linkage disequilibrium (LD) was calculated according to Hill and Weir (1988) using the GenABEL package in R.

A genome-wide association study (GWAS) was carried out in order to detect genetic loci that underlie the difference in the response to the bacterial priming. The total data set of 493,846 SNP markers, phenotypic data by BLUEs, and the q-matrix was used for the GWAS. The GAPIT tool in R was used in which a compressed mixed linear model (cMLM) was applied using q-matrix and kinship (calculated by Van Raden within the package) as random factors (Lipka et al., 2012) after a model selection step including cMLM with kinship as cofactor, a generalized linear model (GLM) and cMLM with q-matrix and kinship as cofactors (**Figure S2**). In this comparison, closest alignment to the line of expected probabilities (black) was shown for cMLM with q-matrix and kinship as cofactors (green, subjacent the black line in the graph), which was therefore selected for GWAS. Furthermore, SNP permutation (1,000 permutations) was applied to assess the empirical significance of SNPs and compression level of 1.9 was used as suggested in the GAPIT package. Significant marker trait associations (MTA) were identified using a threshold of the false discovery rate (FDR) at p<0.05 which in this case represents a likelihood of odds (LOD) score of 4. Significant MTAs were further clustered in corresponding putative QTL by the LD of 21,000 bp. LD was calculated using the R package “GenABEL”. Physical positions together with marker types and the phenotypic variances explained (PVE) are visualized using MapChart program (Voorrips, 2002).

### 2.4 Annotations regarding the Morex reference genome

Annotation of genes at the identified marker positions was obtained from information provided in the Barleymap software module “Locate by position” containing annotations from the Morex genome (Mascher et al., 2017) and updated to the MorexV3 genome released in 2021 (Mascher et al., 2021). Annotation of several iSelect markers was obtained from sequence alignments using the BLAST tool of the UniProt reference database (https://www.uniprot.org/blast/). Gene transcript expression data were obtained from the Expression Atlas database for barley (https://www.ebi.ac.uk/gxa/home#).

## 3 Results

### 3.1 The priming effect varies between the various accessions

An established priming system using *E. meliloti expR*+*ch* as primary trigger was applied (Wehner et al., 2019). The low standard deviation and the associated high repeatability shows that the priming method used, was reproducible.

**Figure 2:**
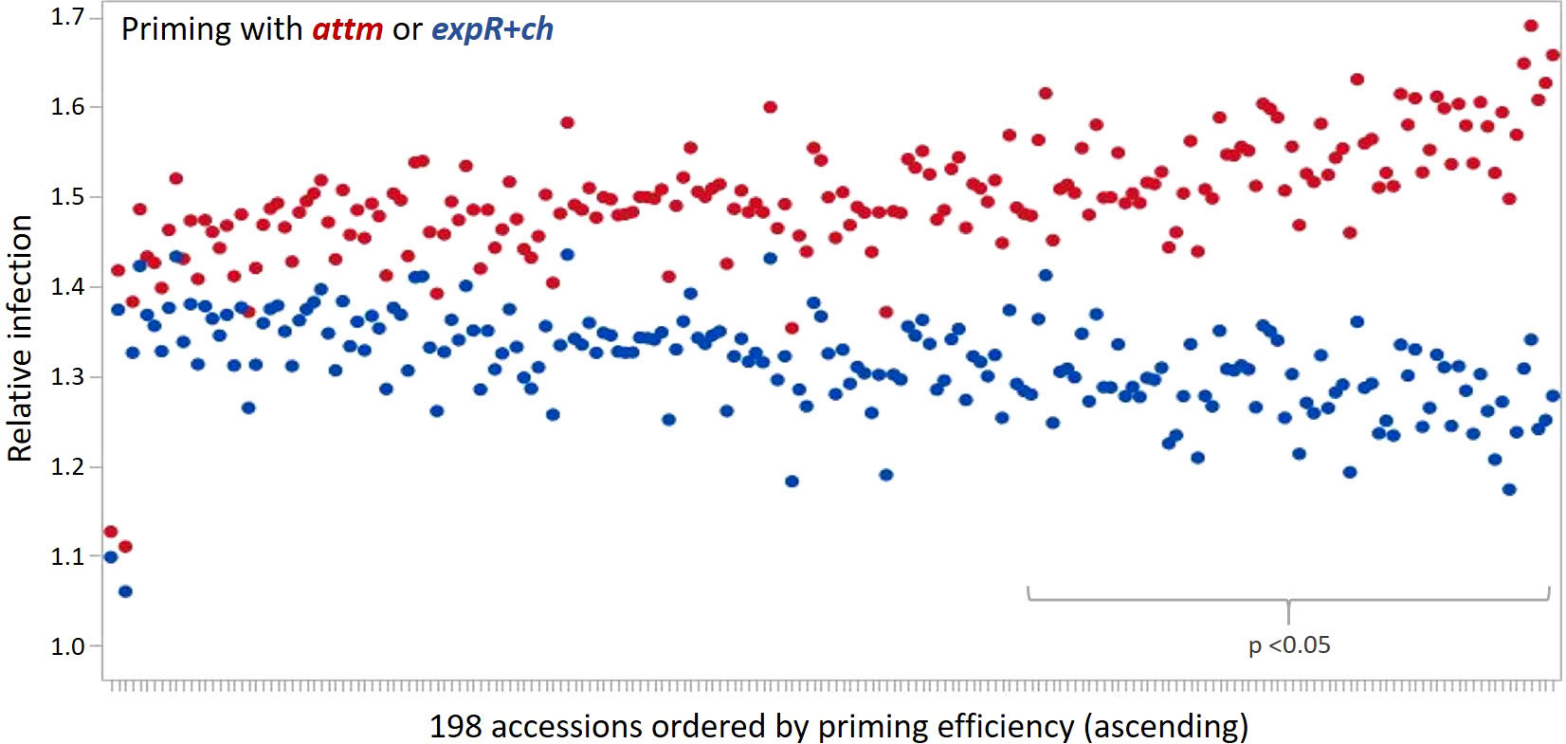
Relative infection with *P. hordei* (BLUES) in the 198 accessions treated with either the *attM* (red) or *expR*+*ch* (blue) bacterial strain and ordered by priming efficiency. Significant treatment effects with p<0.05 were detected for 87 accessions.

The priming effect of the 198 accessions analyzed is represented in the average relative infection with *P. hordei* (**Figure 2**). Results clearly show that the *expR*+*ch* bacterial strain treated group of plants was less infected in comparison to the control group treated with the *attM* bacterial strain (**Figure 2** and **Table 1**). However, a large variation of the accessions regarding the relative infection was observed and allowed for the differentiation in primable and non-primable accessions based on priming efficiency. Significant treatment effects with p<0.05 were detected for 87 accessions meaning that 43.5% of the accessions in our study can be defined as primable with AHL (**Figure 2**, **Table S2**). Concordantly, the large diversity for priming efficiency in the panel is also shown by the high coefficients of variation (CV) for both treatments (**Table 1**). Furthermore, the effects can be assumed stable over the three year’s experiments, with a calculated repeatability of 63.6% for *attm* control treatments, while repeatability was low with 31.4% for the *expR*+*ch* primed plants. In addition, ANOVA results revealed significant effects (p<0.001) of the priming treatment indicating a positive effect of priming on resistance to *P. hordei* infection (**Table 2**). Also, significant (p<0.001) effects between the accessions and for accessions times priming interactions were observed. Thus, from the results a significant priming effect can be concluded for AHL-induced *P. hordei* resistance, which is accession-specific.

The ten accessions with the highest priming efficiency are all of non-European origin, and comprise eight six-rowed and two two-rowed accessions (**Table S2**): BCC129 (MAR, 6-row), HOR11403 (IND, 6-row), BCC421 (CHN. 6-row), BCC551 (IND, 6-row), BCC538 (IND, 6-row), BCC93 (IRQ, 2-row), Morex (USA, 6-row), BCC868 (MEX, 6-row), BCC903 (CAN, 2-row), and BCC881 (CAN, 6-row). Among the ten accessions with the lowest priming efficiency are two of European origin, and four accessions are of six-rowed and six of two-rowed ear type: HOR8160 (TUR, 2-row), BCC192 (SYR, 2-row), BCC899 (CHL, 2-row), HOR11373 (ISR, 2-row), BCC1385 (POL, 2-row), BCC927 (PER, 6-row), BCC445 (CHN, 6-row), BCC844 (COL, 6-row), BCC1433 (DEU, 2-row), and BCC807 (URY, 6-row). However, apart from a trend to non-European origin and six-rowed ear type being correlated with higher priming efficiency no clear relation to genetically determined phenotypic traits could be made.

### 3.2 GWAS identified markers for relative infection

**Figure 3:**
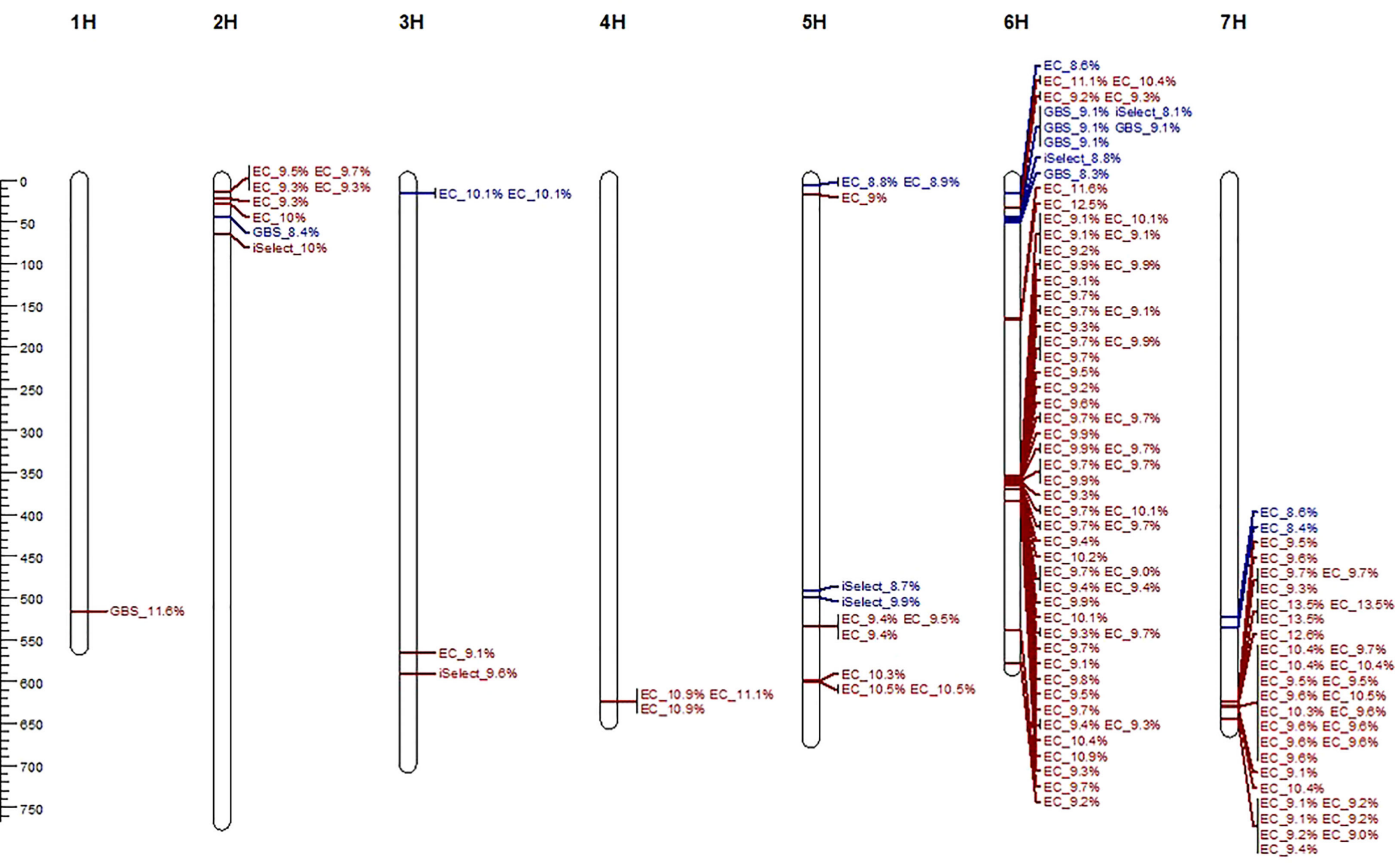
Physical map (positions in Mb) of the seven barley chromosomes and 70 identified quantitative trait loci (QTL) for relative infection with *P. hordei* upon *attM* (red) or *expR*+*ch* (blue) priming, including information on marker type and explained phenotypic variance of each SNP.

For the three-year data on relative infection of primed (*expR*+*ch*) and control plants (*attM*), 129 significant marker-trait associations (MTAs) were identified by GWAS (**Table S3**, **Figure S3**). Notably, primarily exome capture (EC) markers were associated, highlighting the predominance of this marker type in our dataset. Identified MTAs were clustered into QTL using the critical LD decay value of 21,000 bp and MTA not in LD were considered as an independent QTL. Overall, 70 QTL were identified (**Table S3**). The identified QTL were unevenly distributed across the barley chromosomes with one QTL detected on chromosome 1H, five on chromosome 2H, three on chromosome 3H, one on chromosome 4H, seven on chromosome 5H. A hot spot of 42 QTL was detected on chromosome 6H, and 11 QTL were detected on chromosome 7H (**Figure 3**). Detected QTL showed a positive effect on relative infection with significant LOD values ranging between 4.01 and 6.21 (**Table S3**). The lowest LOD value was found for MTA 33 in QTL 21 on chromosome 6H and the highest LOD value was found for MTA 102, 103 and 104 in QTL 65 on chromosome 7H. The respective QTL explain up to 13.5% of the phenotypic variance (PVE) as specified in **Figure 3** and listed in **Table S3**, indicating the potential to increase resistance to *P. hordei* in spring barley.

We have not detected QTL overlapping for relative infection of primed (*expR*+*ch*) and control plants (*attM*) in our study. Of all identified significant MTA, 112 MTA clustered in 59 QTL, which were associated with the relative infection under *attm* treatment. Therein, the percentage of PVE by each MTA varied from 9.0 to 13.5% (**Figure 3**). In addition, 17 MTA clustered in 11 QTL, which are associated with the relative infection under *expR*+*ch* priming (**Table 3**). These 11 QTL were distributed over the barley chromosomes 2H (one QTL), 3H (one QTL), 5H (three QTL), 6H (four QTL), and 7H (two QTL). Variation of PVE by each MTA varied between 8.3 to 10.1% (**Figure 3**).

**Table 3:** In a genome‐wide association study (GWAS), based on the observed phenotypic differences and 493,846 filtered SNPs derived from the Illumina 9k iSelect (iSelect) chip, genotyping by sequencing (GBS), and exome capture data (EC), 11 quantitative trait loci (QTL) associated to improved resistance to *P. hordei* after priming with *E. meliloti expR*+*ch*, were identified with a peak on the short arm of the barley chromosome (Chr.) 6H.

QTL	SNP	Marker type	Chr.	Position in Mb	LOD	Annotation (Barleymap, ^a^UniProt BlastX)	Gene Name	Differential Expression (up-regulated)	Location	GO	InterPro
5	chr2H_44543906	GBS	2H	44,543906	4,13	WRKY transcription factor 4	HORVU2Hr1G017720	Infection, wound	Nucleus	DNA-binding, transcription factor activity	WRKY domain
7	chr3H_15470342EC	EC	3H	15,470342	4,84	Xylanase inhibitor	HORVU3Hr1G006440	Infection, wound		Aspartic-type endopeptidase activity, proteolysis	Xylanase inhibitor, Aspartic peptidase A1
	chr3H_15470343EC	EC	3H	15,470343	4,84	Xylanase inhibitor	HORVU3Hr1G006440	Infection, wound		Aspartic-type endopeptidase activity, proteolysis	Xylanase inhibitor, Aspartic peptidase A1
11	chr5H_5584131EC	EC	5H	5,584131	4,30	Laccase	HORVU5Hr1G001990			Lignin catabolic process	Reverse transcriptase-like
	chr5H_5584216EC	EC	5H	5,584216	4,35	Laccase	HORVU5Hr1G001990			Lignin catabolic process	Reverse transcriptase-like
13	SCRI_RS_205235	iSelect	5H	491,233648	4,25	ATP-dependent zinc metalloprotease FtsH 2^a^	HORVU5Hr1G063340	Infection, wound	Integral membrane	ATPase activity, ATP binding	ATPase, nucleoside triphosphate hydrolase
14	BOPA1_8215-496	iSelect	5H	498,937856	4,73	PDZ domain-containing protein^a^	HORVU5Hr1G065220		Nucleus, cytoplasm	Proteasome regulatory particle assembly, protein binding	26S Proteasome non-ATPase regulatory subunit 9
18	chr6H_16152203EC	EC	6H	16,152203	4,23	Pentatricopeptide repeat-containing protein	HORVU6Hr1G008870	Drought		Nucleic acid binding, protein binding	Zinc finger C2H2 superfamily
21	SCRI_RS_182367	iSelect	6H	44,243728	4,01	Esterase/lipase/thioesterase family protein	HORVU6Hr1G018050		ER membrane	Transferase activity, transferring acyl groups other than amino-acyl groups	Alpha/Beta hydrolase, Diacylglycerol acyltransferase
	chr6H_44247682	GBS	6H	44,247682	4,44	Esterase/lipase/thioesterase family protein	HORVU6Hr1G018050		ER membrane	Transferase activity, transferring acyl groups other than amino-acyl groups	Alpha/Beta hydrolase, Diacylglycerol acyltransferase
	chr6H_44247749	GBS	6H	44,247749	4,44	Esterase/lipase/thioesterase family protein	HORVU6Hr1G018050		ER membrane	Transferase activity, transferring acyl groups other than amino-acyl groups	Alpha/Beta hydrolase, Diacylglycerol acyltransferase
	chr6H_44247752	GBS	6H	44,247752	4,44	Esterase/lipase/thioesterase family protein	HORVU6Hr1G018050		ER membrane	Transferase activity, transferring acyl groups other than amino-acyl groups	Alpha/Beta hydrolase, Diacylglycerol acyltransferase
	chr6H_44247776	GBS	6H	44,247776	4,44	Esterase/lipase/thioesterase family protein	HORVU6Hr1G018050		ER membrane	Transferase activity, transferring acyl groups other than amino-acyl groups	Alpha/Beta hydrolase, Diacylglycerol acyltransferase
22	SCRI_RS_121633	iSelect	6H	47,62853	4,33	Protein FLUORESCENT IN BLUE LIGHT	HORVU6Hr1G018610		Chloroplast membrane	Chlorophyll/tetrapyrrole biosynthetic process, protein binding	Protein FLUORESCENT IN BLUE LIGHT, Tetratricopeptide repeat-containing
23	chr6H_50004267	GBS	6H	50,004267	4,11						
60	chr7H_523570079EC	EC	7H	523,570079	4,25	Undescribed protein	HORVU7Hr1G086760				
61	chr7H_535632749EC	EC	7H	535,632749	4,17						

Positions of the detected SNP marker were mapped against the barley reference genome and identified QTL and detected SNP marker in these regions are listed together with the annotation and available gene transcript expression information.

Out of the 70 detected QTL, annotation of marker positions within 23 QTL could not be obtained. This relates to 34 of the 129 identified MTA (**Table S3**). From the annotated genes, 13 are related to proteins with unknown functions (unknown protein or undescribed protein). Promising candidates associated with increased resistance to *P. hordei* treated with the *attm* control strain included i) several transporters: Magnesium transporter NIPA2 (HORVU1Hr1G077020) in QTL one, Mechanosensitive ion channel protein 2 (HORVU5Hr1G095750) in QTL 17, LETM1 and EF-hand domain-containing protein 1 (HORVU6Hr1G055750) in QTL 28, ii) transcription factors: WRKY DNA-binding protein 2 (HORVU5Hr1G072020) in QTL 16, MYB-like 102 (HORVU6Hr1G058640) in QTL 58, and iii) several proteins involved in nucleic acid and protein processing (**Table S3**). Unfortunately, the three markers with the highest LOD value of 6.21 (MTA 102, 103 and 104) in QTL 65 on chromosome 7H could not be annotated, yet.

The most interesting QTL associated with increased resistance to *P. hordei* primed with *E. meliloti expR*+*ch* was identified on the short arm of barley chromosome 6H (**Figure 3**, **Table 3**). The five marker clustering into QTL 21 are located in the gene HORVU6Hr1G018050 being annotated as esterase/lipase/thioesterase family protein. In addition, the two markers clustering into QTL 7 on chromosome 3H, are located in the gene HORVU3Hr1G006440 which is a xylanase inhibitor. This gene has been reported to show increased transcript accumulation under infection and wounding (**Table 3**). The former is also the QTL containing the markers with the highest PVE among the QTL associated with increased resistance to *P. hordei* after AHL-*priming*, with 10.1% (**Figure 3**). Similarly, the two markers in QTL 5 and QTL 13 are located in genes, which show increased transcript expression under infection and wounding: namely HORVU2Hr1G017720, the WRKY transcription factor 4, and HORVU5Hr1G063340, the ATP-dependent zinc metalloprotease FtsH 2, respectively. Also, HORVU5Hr1G001990, a laccase involved in lignin catabolism, may be involved in increased resistance to *P. hordei* following AHL-*priming*. The two markers clustering into QTL 11 on chromosome 5H were located in this gene (**Table 3**).

## 4 Discussion

Within this three-year trial, a positive effect of priming with the gram negative soil bacterium *E. meliloti* strain *expR*+*ch* in relation to resistance to *P. hordei* was demonstrated for spring barley. The effect on relative infection and thus priming efficiency varied among the set of 198 accessions, of which 87 accessions showed significant (p<0.05) treatment effects and therefore have been considered highly primable (**Figure 2**). Nevertheless, in practical applications the priming effect might be influenced by environmental conditions, such as soil type and the presence of other microorganisms. To our knowledge, the presented study is the first investigation on the effects of the priming inducer *E. meliloti* strain *expR*+*ch* on cereal resistance against fungal pathogens including a larger set of genetically differentiating accessions. While several recent reports are suggesting that the microbiome varies between the different plant organs, developmental stages, origin, as well as between genotypes in wheat (Gdanetz and Trail, 2017; Kuźniar et al., 2020; Žiarovská et al., 2020) and barley (Yang et al., 2017; Alegria Terrazas et al., 2020; Bziuk et al., 2021), studies on genetic variation of priming efficiency are rare. In a previous experiment, we observed significant differences between seven spring barley accessions, indicating genotypic differences in the response to priming by *E. meliloti* strain *expR*+*ch* towards *P. hordei* resistance (Wehner et al., 2019). Similarly, using a set of eight genetically diverse spring barley accessions, Shrestha et al. (2019) have demonstrated differences in their ability to be primed by *E. meliloti* strain *expR*+*ch* with respect to resistance against *B. graminis* f. sp. *hordei*. Other root associated microorganisms, for which genotype dependent responses have been shown, include arbuscular mycorrhiza fungi (AMF), reviewed in Parke and Kaeppler (2000) and Sawers et al. (2010). Varying abilities to respond to AMF were reported for wheat (Hetrick et al., 1993), maize (Kaeppler et al., 2000; An et al., 2010), and onion (Powell et al., 1982; Tawaraya et al., 2001; Galván et al., 2011). Consistently, investigation of a diverse set of 94 bread wheat genotypes (*Triticum aestivum*) regarding root colonization by AMF identified significant genotypic differences (p<0.001) for mycorrhizal colonization and detected 30 significant genetic markers associated with root colonization (Lehnert et al., 2017). Within the same set of genotypes, drought stress tolerance was significantly increased in the presence of AMF colonization compared to drought stress tolerance in the absence of AMF colonization (Lehnert et al., 2018).

In our results, we observed a trend to non-European origin and six-rowed ear type being correlated with higher priming efficiency (**Table S2**). However, no clear relation to genetically determined phenotypic traits could be made. The model cultivar Morex was among the ten accessions with highest priming efficiency. This is concordant with our previous finding, where Morex was identified as a well primable accession (Wehner et al., 2019). In this study, the accessions BCC768 and HOR7985 were also assigned as well-primable, a result which was confirmed in the recent study. While the resistance gene *Rph8* was mapped in the Morex reference genome on chromosome 5H at 12.7 Mb (Mascher et al., 2017), we observed high values for relative infection under *attM* control treatment in this accession (**Table S2**). This can be explained by breakdown of resistance gene *Rph8* by the *P. hordei* I-80 isolate (Niks et al., 2000), which was used in the present study. Notably, some QTL identified during the control treatment were in the genomic region of known resistance genes, for example *Rph19* (Park and Karakousis, 2002) on the barley chromosome 7H (see **Figure 3**). Together, results suggest a high genetic determination of priming responses and thus highlight the importance of screening large diversity sets for detecting quantitative trait loci or candidate genes involved in priming.

In our study, of the 129 MTA identified, 17 MTA, which cluster in 11 QTL, were associated to improved resistance to *P. hordei* after priming with the oxo-C14-HSL-producing *E. meliloti expR*+*ch*, with a hot spot on the short arm of barley chromosome 6H (**Figure 3**, **Table 3**, and **Table S3**). Except two markers, all markers were assigned to positions in the genome within annotated barley coding genes. On chromosome 6H, the marker chr6H_16152203EC (QTL 18, LOD 4.23) is localized in the gene HORVU6Hr1G008870 encoding for a pentatricopeptide repeat-containing protein. This gene product belongs to the class of zinc finger proteins involved in DNA binding, which may have regulatory function, and which was shown to be induced by drought stress in barley in a previous study (Guo et al., 2009). Another significant marker on chromosome 6H (SCRI_RS_121633, QTL 22, LOD 4.33) is localized in the gene HORVU6Hr1G018610 encoding for the protein FLUORESCENT IN BLUE LIGHT. In *A. thaliana*, this gene product was reported as a negative regulator of chlorophyll biosynthesis acting in a light dependent manner (Hou et al., 2019). How both gene products mentioned above are involved in the process of induced systemic resistance by AHL priming remains speculation. However, limitation of photosynthetic activity has been assumed one possible strategy of plants to increase pathogen resistance. For instance, the accumulation of oxylipin in distal tissues during AHL-priming in Arabidopsis promoted stomatal closure. The closed stomata enhanced plant resistance to bacterial pathogens (Schenk and Schikora, 2015). In this line, a very promising QTL (21) on chromosome 6H comprises five significant markers from two different marker types (SCRI_RS_182367, chr6H_44247682, chr6H_44247749, chr6H_44247752, chr6H_44247776), all localized in the same gene HORVU6Hr1G018050, which encodes for a esterase/lipase/thioesterase family protein. In barley, this protein was not characterized functionally yet, but a BLAST search with the protein sequence in the NCBI database (https://blast.ncbi.nlm.nih.gov/Blast.cgi) revealed highest similarity to the chloroplastic phytyl ester synthase 1 (PES1) from *A. thaliana* (At1g54570). It exhibits phytyl ester synthesis and diacylglycerol acyltransferase activities with broad substrate specificities, and can employ acyl-CoAs, acyl carrier proteins, and galactolipids as acyl donors. PES1 was shown to be involved in fatty acid phytyl ester synthesis in chloroplasts, a process required for the maintenance of the photosynthetic membrane integrity during abiotic stress and senescence (Lippold et al., 2012). Accordingly, deposition of free phytol and free fatty acids in the form of phytyl esters in chloroplasts was reported for etiolated barley seedlings after 6 to 8 h irradiation (Liljenberg, 1977), and in primary leaves of barley during methyl jasmonate induced leaf senescence (Springer et al., 2015). Notably, HORVU6Hr1G018050 was also found in a QTL region on chromosome 6H in the interval between 37-76 Mb associated with resistance against net blotch causing *Pyrenophora teres* f. *teres* in a barley diversity set (Novakazi et al., 2019). On chromosome 5H, the marker BOPA1_8215-496 (QTL 14, LOD 4,73) is localized in the gene HORVU5Hr1G065220 encoding for a PDZ domain-containing protein, which is likely involved in the regulation of protein translation *via* the proteasome with unknown contribution to pathogen resistance. Interestingly, the marker SCRI_RS_205235 (QTL 13, LOD 4,25) is localized in the ATP-dependent zinc metalloprotease FtsH coding gene 2HORVU5Hr1G063340. FtsH is the major thylakoid membrane protease required for photosynthetic pathways in plants (Kato and Sakamoto, 2018). This gene was recently shown to be relatively highly expressed in all grain organs suggesting its crucial role in the accumulation of the micronutrient metals (i.e., Cu, Fe, Mn, and Zn) in barley grains (Thabet et al., 2022). It may be speculated, that FtsH represents another component of the mechanisms activated to limit photosynthetic activity to increase pathogen resistance. However, despite the upregulation observed during infection and wounding (**Table 3**), the involvement of FtsH in improved resistance against *P. hordei* after AHL-priming needs further molecular validation. Two other markers on chromosome 5H (chr5H_5584131EC, chr5H_5584216EC) cluster in QTL 11 with LOD 4.3 and 4.35, respectively. Both are located in the same gene HORVU5Hr1G001990, which encodes for a laccase. Laccases constitute a multi-gene family of multi-copper glycoproteins and have diverse and overlapping physiological functions in plants, including involvement in redox metabolism, responses to wound healing, defense against pathogens or insects, synthesis of lignin and suberin, and cross-linking of cell wall components; cf. Janusz et al. (2020) and Tomková et al. (2012). While several laccases have been annotated in the barley genome on all chromosomes recently (https://plants.ensembl.org/Hordeum_vulgare/Search/Results?page=3;q=laccase;species=Hordeum_vulgare;collection=all;site=ensembl), only *HvLac1* on chromosome 4H has been characterized in detail so far with unclear molecular function (Tomková et al., 2012). However, we suggest that the laccase, identified in our study is likely involved in cell wall fortification representing one of the processes leading to increased resistance to *P. hordei* by AHL priming. Consistently (Shrestha et al., 2019) showed that the principal mechanism of AHL-induced priming in barley seems similar to the mechanism in Arabidopsis, i.e., it is associated with the activation of MAPKs, enhanced expression of various defense-related genes and of genes involved in the remodeling of cell wall structure. Additionally, laccases were also identified among a group of genes that were associated with *Fusarium graminearum* resistance in barley (Huang et al., 2016). In the same line, the two markers identified on chromosome 3H (chr3H_15470342EC, chr3H_15470343EC, QTL7, LOD 4.84), which are both localized in the gene (HORVU3Hr1G006440) annotated as a xylanase inhibitor, represent interesting candidates with potential function in the fortification and remodeling of cell wall structure. This gene was also found to be upregulated after infection and wounding in barley (**Table 3**). Most cereals contain arabinoxylan as a structural component in their cell walls (Henry, 1985). As xylanases have a general role in the depolymerization of plant and fungal cell walls (Hrmova et al., 1997), the xylanase inhibitor identified in our study could help to reduce or overcome the effect of plant and fungal related xylanases during infection with *P. hordei* and thus promote resistance. A marker with high potential in translation regulatory processes was identified on chromosome H2 (chr2H_44543906, LOD 4.13) localized in the gene HORVU2Hr1G017720, which is annotated as WRKY transcription factor 4. The WRKY transcription factor gene family is one of the largest, and it is known to be involved in a wide range of plant developmental and physiological processes. Although, more than 60 unique barley genes have been annotated containing the WRKY domain, WRKY proteins of barley are not yet fully annotated and most of them are not functionally characterized (Liu et al. (2014), http://www.barleygfdb.com/second/family.php?xu=77). The data of Dey et al. (2014) suggest that bacteria-induced systemic immunity in barley is associated with the local and/or systemic induction of transcript accumulation of Ethylene Responsive Factor (ERF)-like transcription factors (HvERF-like, HvERF44411) as well as HvWRKY22 and HvWRKY38/1. Similarly, Gao et al. (2018) proposed Nonexpressor of Pathogenesis-Related Genes1 (NPR1) homologs and WRKY transcription factors as the master regulators of systemic acquired resistance in wheat and barley. Furthermore, they showed that transient expression of HvWRKY6, HvWRKY40, and HvWRKY70, in wheat leaves by *Agrobacterium*-mediated infiltration enhanced the resistance to *Puccinia triticina*. In another study, HvWRKY10, HvWRKY19, and HvWRKY28 were shown to positively regulate the response of barley to *B. graminis* infection (Meng and Wise, 2012). On the other hand, the wound and pathogen-inducible HvWRKY1 and HvWRKY2 are known as negative defense regulators repressing the activity of the powdery mildew-induced promoter of HvGER4c, a germin-like defense-related protein (Liu et al., 2014). However, WRKY transcription factor 4 (HORVU2Hr1G017720) was not functionally characterized yet, despite the induced expression of HORVU2Hr1G017720 after fungal infection and wounding (**Table 3**, http://www.barleygfdb.com/second/PRJNA728113.php?xu=77). It also needs to be considered that WRKY functions may be genotype specific, such as shown for WRKY transcription factors in barley cultivars infected with *Fusarium culmorum* (Uluhan et al., 2019).

In conclusion, our results provided clear evidence for the improved resistance of barley against *P. hordei* infection by AHL-mediated priming. Priming responses were genotype specific and most significant genetic markers for improved resistance to *P. hordei* after priming identified in our study were related to limiting photosynthetic activity, cell wall fortification, and regulation of transcription and translation. Our findings are important, as they open doors to study the mechanisms and the interactions between the plant genetic background and AHL-priming as well as supporting novel breeding approaches for priming efficient accessions and thus sustainable crop protection after validation.

## Data availability statement

The original contributions presented in the study are included in the article/Supplementary Material. Further inquiries can be directed to the corresponding author.

## Author contributions

The initial research idea was developed by FO and GW. The study conception and experimental design was established by AS and GW. Material preparation and data collection was performed by GW, and data analysis was performed by GW and AM. The first draft of the manuscript was written by GW, amended by AM, and all authors commented on previous versions of the manuscript. All authors read and approved the final manuscript.

## Supplementary material

Supplementary Figure 1 Composition of the test panel of 200 spring barley accessions. Our study panel consisted of 111 two-rowed and 83 six-rowed accessions from the Genobar panel (Haseneyer et al., 2010; Pasam et al., 2012) comprising barley accessions and landraces of worldwide origin and including a test set of six common barley lines. * The UN 3-letter country code was used.

Supplementary Figure 2 QQ-Plot for model selection of a compressed mixed linear model (cMLM, **A**) with kinship as cofactor, cMLM with q-matrix and kinship as cofactors **(B)** and a generalized linear model (GLM, **C**).

Supplementary Figure 3 Manhattan plots for relative infection of *P. hordei* for 200 accessions primed with *E. meliloti* strain attM (red) or expR+ch (blue) against physical positions (Mb) of the marker trait associations on the seven barley chromosomes.
