# Corrigendum: QTL for induced resistance against leaf rust in barley

**DOI:** 10.3389/fpls.2025.1584392

**Published:** 2025-05-26

**Authors:** Andrea Matros, Adam Schikora, Frank Ordon, Gwendolin Wehner

**Affiliations:** ^1^ Julius Kühn Institute (JKI), Federal Research Centre for Cultivated Plants, Institute for Resistance Research and Stress Tolerance, Quedlinburg, Germany; ^2^ Julius Kühn Institute (JKI), Federal Research Centre for Cultivated Plants, Institute for Epidemiology and Pathogen Diagnostics, Braunschweig, Germany

**Keywords:** biotic stress resistance, Ensifer meliloti, AHL priming, Puccinia hordei, barley, leaf rust, GWAS, QTL

In the published article, there was an error in the formula for calculation of Relative infection.

A correction has been made to **2. Material and methods**, *2.2. Phenotyping for relative infection and priming efficiency.* The formula previously stated:

“Relative infection = 0.2 x ln (P. hordei %) + (P. hordei scores)”

The corrected sentence appears below:

“Relative infection = 0.2 x ln (P. hordei %) + ln (P. hordei scores)”

The authors apologize for this error and state that this does not change the scientific conclusions of the article in any way. The original article has been updated.

