# Supplementary figures and images for: QTL for induced resistance against leaf rust in barley
Source: Front Plant Sci. 2023 Jan 12;13:1069087. doi: 10.3389/fpls.2022.1069087 (PMC9877528; doi:10.3389/fpls.2022.1069087)

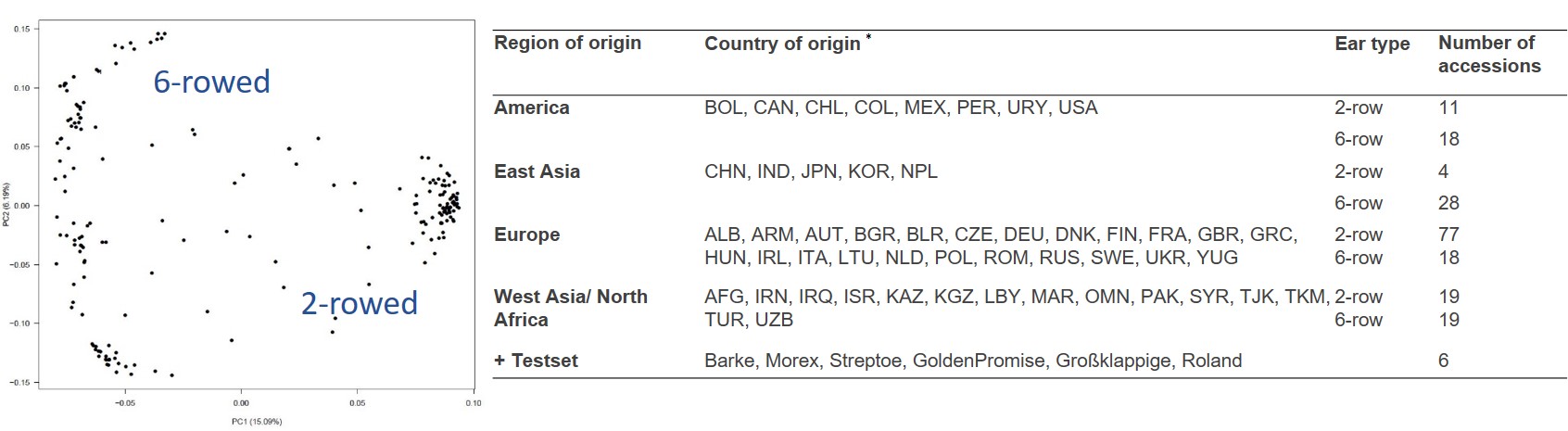

Supplement: Supplementary Figure 1 — Composition of the test panel of 200 spring barley accessions. Our study panel consisted of 111 two-rowed and 83 six-rowed accessions from the Genobar panel (Haseneyer et al., 2010; Pasam et al., 2012) comprising barley accessions and landraces of worldwide origin and including a test set of six common barley lines. * The UN 3-letter country code was used. [file Image_1.jpeg]

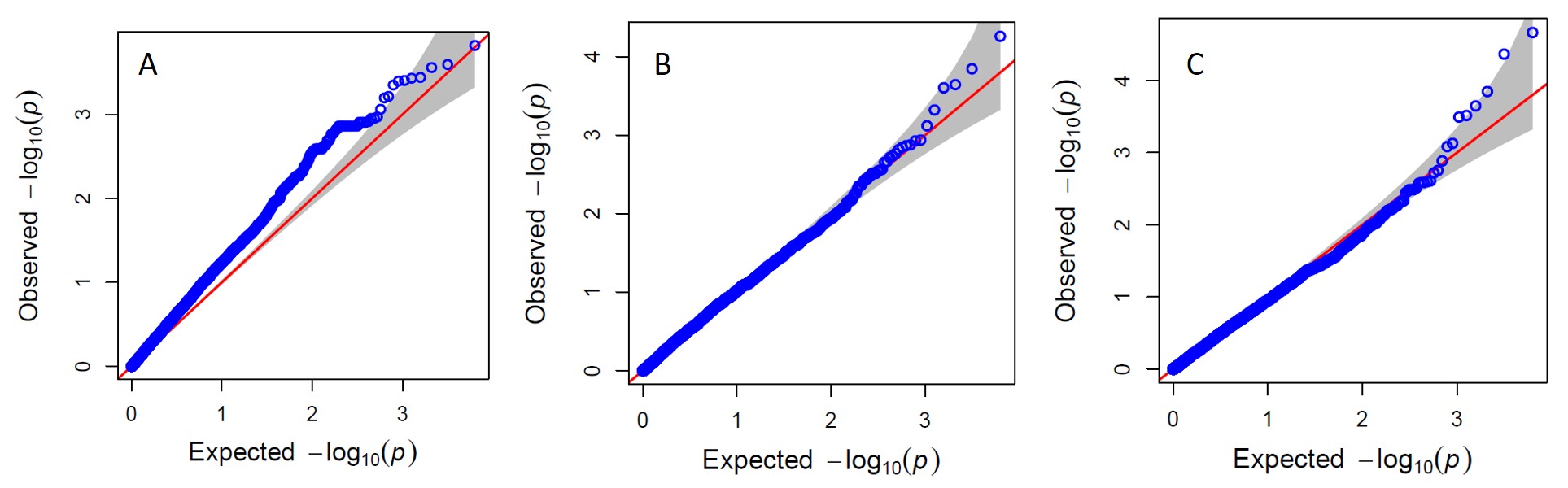

Supplement: Supplementary Figure 2 — QQ-Plot for model selection of a compressed mixed linear model (cMLM, A) with kinship as cofactor, cMLM with q-matrix and kinship as cofactors (B) and a generalized linear model (GLM, C). [file Image_2.jpeg]

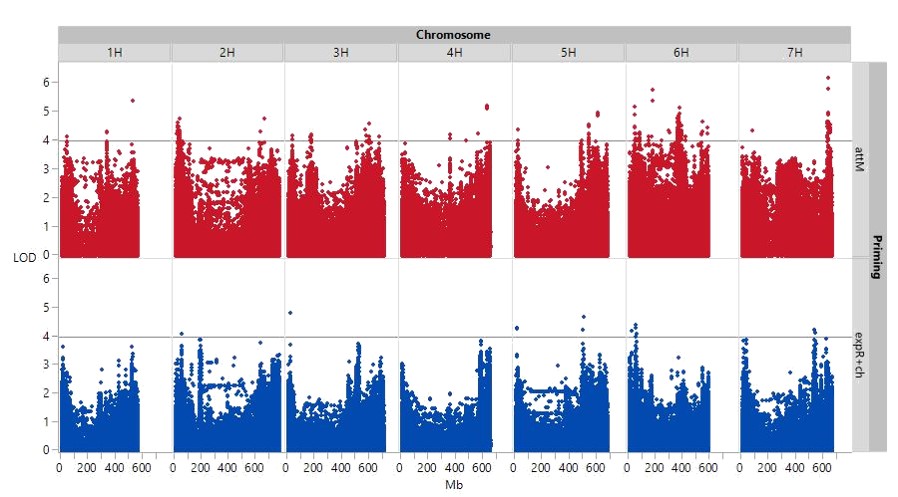

Supplement: Supplementary Figure 3 — Manhattan plots for relative infection of P. hordei for 200 accessions primed with E. meliloti strain attM (red) or expR+ch (blue) against physical positions (Mb) of the marker trait associations on the seven barley chromosomes. [file Image_3.jpeg]
